# The pathway to diagnosis and follow-up care for atrial fibrillation in Sri Lanka: a descriptive longitudinal study

**DOI:** 10.3310/nihropenres.13497.1

**Published:** 2023-11-24

**Authors:** Vethanayagam Antony Sheron, Tiffany E. Gooden, Powsiga Uruthirakumar, Kanesamoorthy Shribavan, Mahesan Guruparan, Kumaran Subaschandren, Gregory Y. H. Lip, Krishnarajah Nirantharakumar, G. Neil Thomas, Rajendra Surenthirakumaran, Balachandran Kumarendran, Semira Manaseki-Holland, NIHR Global Health Research Group on Atrial Fibrillation Management

**Affiliations:** 1 Department of Community and Family Medicine, Faculty of Medicine, University of Jaffna, Jaffna, Northern Province, 40000, Sri Lanka; 2 Institute of Applied Health Research, University of Birmingham, Birmingham, England, B15 2TT, UK; 3 Department of Cardiology, Teaching Hospital Jaffna, University of Jaffna, Jaffna, Northern Province, 40000, Sri Lanka; 4 Liverpool Centre for Cardiovascular Science at University of Liverpool, Liverpool John Moores University, Liverpool, England, L2 2QP, UK; 5 Danish Center for Health Services Research, Department of Clinical Medicine, Aalborg Universitet, Aalborg, North Denmark Region, DK - 9100, Denmark

**Keywords:** atrial fibrillation, anticoagulation, preventative treatment, follow-up care, INR, COVID-19

## Abstract

**Background:**

Early diagnosis and continuity of care is vital for atrial fibrillation (AF), a major risk factor for stroke that requires regular monitoring when treated with warfarin; however, evidence on AF care in LMICs is lacking. We aimed to identify the AF patient pathway in Northern Province, Sri Lanka and to determine how the COVID-19 pandemic impacted AF care.

**Methods:**

This descriptive longitudinal study utilised two questionnaires, which were previously used in India and Mongolia to quantitatively evaluate the AF pathway: one at baseline and one ≥3 months following baseline. Adults (≥18 years) with AF were recruited from the A&E department and outpatient clinics located at the Jaffna Teaching Hospital, the only tertiary hospital with cardiologists and 12-lead echocardiogram facilities in the Province. Data were collected between October 2020 and June 2021 and analysed using descriptive statistics.

**Results:**

This study included 151 participants (median age 57 years, IQR 49-67; 70% female). Most participants were diagnosed in the A&E (38%) or inpatient department (26%), followed by an outpatient department (19%) or private facility (16%). Nearly all (97%) participants received follow-up care during the study period, with an average of 1.3 AF-related healthcare visits per person; most visited an outpatient department (88%). The COVID-19 pandemic negatively impacted 39% of participants’ care: fewer healthcare visits, delayed or unattainable medications, and longer intervals between blood tests; however, 24% of participants received their medication by ambulance, public health staff or post.

**Conclusions:**

Primary care was not involved in the diagnosis of AF, leading to most diagnoses occurring after a medical emergency. The frequency of blood tests was lower than guideline recommendations and could in-part be due to the adverse impacts of the pandemic. Strengthening primary and community-based care may enable early diagnosis and improve continuity of care during and beyond future healthcare crises/emergencies.

## Plain english summary

Atrial fibrillation (AF) is a condition that affects the heart rhythm and increases the risk of stroke. AF can be treated by anticoagulant drugs, which thin the blood and prevent blood clots. Warfarin, the most common anticoagulant drug prescribed for AF in low- and middle-income countries (LMICs), is effective in controlling the heart rhythm and can reduce strokes. However, the dose of warfarin must be monitored monthly (typically by a blood test called international normalised ratio (INR)) and often changed to ensure patient safety. It is important for AF to be diagnosed early within primary care for people to receive life-saving medication to effectively manage AF and prevent stroke.

Sri Lanka, a LMIC in Southeast Asia, has limited healthcare resources and poor awareness of AF. Like most countries around the world, the coronavirus disease (COVID-19) pandemic impacted healthcare in Sri Lanka with travel restrictions and lockdowns; AF care was likely impacted but, to the best of our knowledge, no study has investigated how. Therefore, we administered a questionnaire to people with AF receiving care from the only teaching hospital in Northern Province Sri Lanka that has a specialised clinic for AF aimed at understanding where people with AF get diagnosed, where they receive follow-up care and how the COVID-19 pandemic impacted their care.

None of the 151 participants were diagnosed in primary care. Most participants were diagnosed in emergency or inpatients departments, indicating that AF was recognised after a major health problem such as stroke. The frequency of follow-up visits during the study period to receive an INR test was lower than guideline recommendations and the COVID-19 pandemic negatively impacted the availability and accessibility of warfarin and INR tests.

This study suggests that primary care must be strengthened to enable early diagnosis and improve continuity of care, especially during healthcare crises like a pandemic.

## Introduction

Atrial fibrillation (AF) is the most common cardiac arrhythmic condition worldwide, affecting more than 37 million people ^1, 2^. AF has a major public health impact due to the increased risk of hospitalisation and stroke in patients with AF ^2^. Based on the 2017 Global Burden of Disease Study, there are 2.9 million new AF cases a year on average ^3^. A meta-analysis of 29 trials in patients with AF revealed that the use of adjusted-dose warfarin, a commonly prescribed oral anticoagulant (OAC), reduced stroke by 64% and all-cause mortality by 26%, when compared to placebo or control ^4^. However, AF-related stroke is still frequently overlooked in low- and middle-income countries (LMICs), with underutilised OAC use, missed opportunity for treating underlying risk factors ( *e.g.*, hypertension, diabetes, cardiovascular risks) and various challenges for continuous monitoring of warfarin dose ^4^. Preventing and optimising treatment for AF will aid in reaching the ambitious Sustainable Development Goal 3, which aims to reduce the mortality rate due to cardiovascular diseases by one-third by year 2030 ^5^.

The Atrial fibrillation Better Care (ABC) pathway ^6^ is recommended for effective AF management; this involves avoiding stroke with anticoagulation therapy, better management of symptoms with rate or rhythm control and management of cardiovascular risk, comorbidities and lifestyle behaviours. Well-designed cohort studies ^7– 10^ and one clinical trial ^11^ have confirmed that this integrated approach can lower the risk of AF adverse outcomes, including thromboembolic incidents, and reduce the risk of mortality and hospitalisations from AF ^12^, leading to its recommendations in guidelines ^13, 14^. The ABC pathway is well established in high income countries (HICs) but implementation of the pathway has been limited in LMICs ^15^. Many barriers for implementing the pathway may exist in LMICs; for instance inadequate primary care facilities, poor handover practices, fragmented services, lack of patient and clinician knowledge of AF, access to diagnostic facilities, medication and tests, and adherence to medication, tests and follow-up care ^16– 18^. LMICs also face multiple challenges for optimising the care pathway due to the growing elderly population, limited resources of staff, equipment and funds from Ministries of Health, and general increased prevalence of non-communicable diseases (NCDs) ^19^. For instance, it is projected that in Asia alone, 72 million people will have AF by 2050, among them 2.9 million will suffer AF-related stroke ^20^.

Sri Lanka is a low-income South Asian country recovering from decades of conflict ^21^ and is currently suffering from an economic crisis ^22^. Whilst the country is rapidly reconstructing its healthcare system, evidence is needed on how to ensure optimal management of AF, including early diagnosis, stroke-preventative medication, and reduction of risk factors in normal times and in times of crises. AF may be diagnosed in Accident and Emergency (A&E), inpatient or outpatient departments of tertiary or secondary care hospitals, primary care units or in private care facilities. Once diagnosed and prescribed warfarin, patients may be referred to receive follow-up care, for instance to take international normalised ratio (INR) tests and collect their medications from specialised clinics or secondary care hospitals. However, the current and most common pathway of AF care that patients take is unknown. Understanding how patients with AF navigate through the healthcare system will highlight gaps, inefficiencies and strengths of current care; thus, enabling effective health system changes and implementation of the ABC pathway for the improvement of AF care and management in Sri Lanka, neighbouring South Asian countries and similar LMICs in other regions of the world.

We therefore aimed to identify the AF care pathway in Sri Lanka from the patients’ perspective; more specifically, where patients with AF are diagnosed, where they go for continued AF care and management and to determine the proportion of patients with AF that are taking OACs and routinely receiving INR tests. After the study commenced, the coronavirus disease (COVID-19) pandemic occurred; in response, we added a secondary aim to assess the impact of the pandemic on the AF care pathway.

## Methods

### Patient and public involvement

Patient partners were not involved in the design or conduct of this study.

### Ethics and consent

Ethical approval was received on 8 ^th^ July 2020 in Sri Lanka from the Ethical Review Committee, Faculty of Medicine and University of Jaffna, Sri Lanka (Reference number/ERC/19/ 107/NDR/0218). Written informed consent was provided from all included participants either through signature or thumbprint on paper if illiterate.

### Study design

This study was part of a multi-country study aimed to identify the AF care pathway in Brazil, China and Sri Lanka using a prospective study design ^23^. Adults (≥18 years) with a confirmed diagnosis of AF or an arrhythmia likely to be AF who were visiting any of the included healthcare facilities for AF care and spoke the local language (Tamil) were considered eligible. Patients were excluded if they had any hearing or cognitive impairment.

### Setting

Patients were recruited from the Northern Province in Sri Lanka. The estimated population in the Northern Province is 1.2 million and comprises five districts, including urban, rural and peri-urban settings ^24^. Universal healthcare is available across all of Sri Lanka, with care and medications free to all citizens; however, in the Northern Province, only one tertiary hospital (Jaffna Teaching Hospital) exists with cardiologists and 12-lead echocardiogram facilities. Participants were recruited from Jaffna Teaching Hospital (part of University of Jaffna) within the A&E department, anticoagulation clinic and the medical clinic. Sex differences were not taken into the consideration in the study design as the study primary aim was to understand where people with AF get diagnosed, where they receive follow-up care and how the COVID-19 pandemic impacted their care, which is not impacted by sex differences.

### Data collection

A research fellow with a master’s degree in applied epidemiology and seven trained research assistants with degrees in medicine, Siddha medicine or nursing conducted consecutive recruitment by approaching patients in the A&E department, anticoagulation clinic and medical clinic, inviting all eligible patients to participate. The anticoagulation clinic was visited on Tuesdays (the clinic’s dedicated day for patients on warfarin to conduct INR tests), whereas the medical clinic was visited weekdays and weekends randomly throughout the study period due to the clinic not having a designated day for AF care. If a patient agreed to take part, they provided written informed consent and baseline data was collected during their wait to see the doctor.

Baseline data was completed between October 2020 and March 2021. Follow-up data was completed in person at the specialised clinics or by phone for each participant between April 2021 and June 2021, at least three months after baseline data was collected for each participant. Two questionnaires were developed and adapted from studies in Mongolia ^25^ and India ^26^ to collect quantitative data on AF diagnosis, management and care. The baseline questionnaire aimed to capture how and when an AF diagnosis was received, whereas the follow-up questionnaire aimed to capture patients’ healthcare seeking behaviours and the care and medication received since baseline; the follow-up questionnaire was administered at least two months following the baseline questionnaire. The questionnaires were translated into Tamil, then back translated into English to check for accuracy. Before the study commenced, the questionnaires were piloted with four patients in Sri Lanka; no major changes resulted from piloting the tools ^23^. KoBoCollect, (version v2021.2.4.) an open-source software platform used in many LMICs, was used to collect all data ^27^.

Prior to recruitment, the COVID-19 pandemic occurred, with Sri Lanka experiencing a distinct wave of cases during the recruitment period. During this time, partial or complete lockdowns were implemented and limitations on travel and healthcare provision were imposed across the country. However, clinic visits in the hospital (where patients were recruited) remained normal during the entirety of the study and data collection was feasible following arrangements to maintain social distancing.

To ensure we captured how the aspects of the pathway had changed due to the pandemic, we adapted the follow-up questionnaire to include questions on the impact of COVID-19 on clinic visits, receiving medications and INR tests. The original protocol for the study was amended to add the secondary aim of identifying the impact of the pandemic on the AF care pathway in Sri Lanka to ensure we present findings from the original aims within the context of the emergent pandemic.

### Statistical methods

 IBM SPSS Statistics (RRID:SCR_016479) 27.0 (IBM Corp., Armonk, N.Y., USA) was used for all analyses. Descriptive statistics were used to present baseline and follow-up data. Continuous variables are presented as mean with standard deviation (±SD) and categorical variables are presented as frequencies and proportions.

The sample size calculation was based upon achieving an adequate precision for the primary aim of the study of identifying the number of AF diagnoses in primary care settings. A minimum sample size of n=205 was calculated based on a formula for accurately estimating the proportion in an unknown population with ±5% accuracy at the 95% confidence level (α=0.05) ^25^. After a 15% increase was added to the sample size to account for loss to follow-up, the final required sample size was 236.

## Results

### Participant characteristics

**Figure 1.**
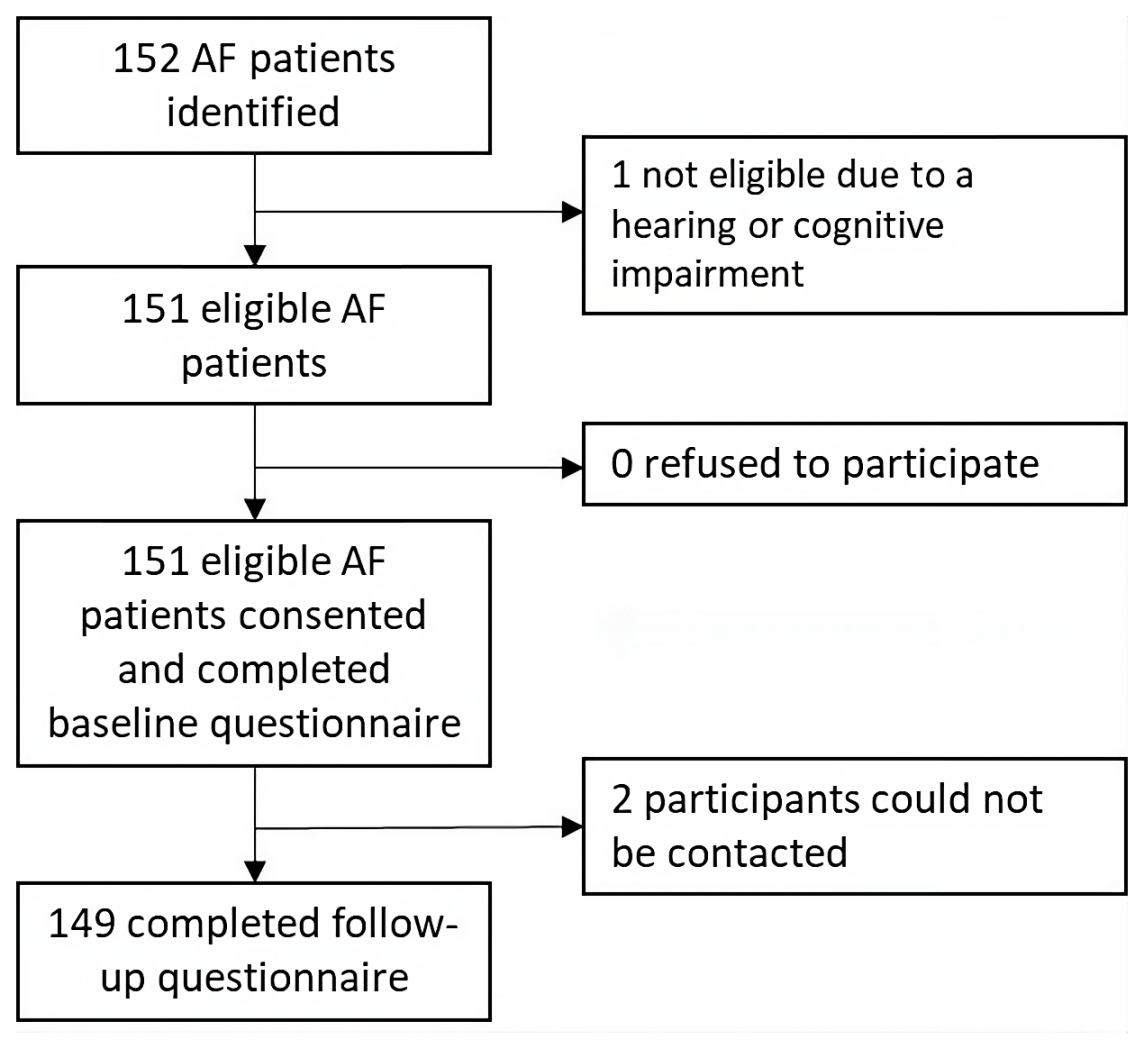
Study flow diagram. AF, atrial fibrillation.

A total of 152 patients with AF were identified from the A&E and outpatients clinics located at the Jaffna Teaching Hospital ( Figure 1). One patient was excluded due to a hearing impairment, though the remaining 151 patients provided consent and were included in the study. Of these, 119 were recruited from the anticoagulation clinic, 30 from the medical clinics and two from the A&E. None of the participants were receiving first-time AF care. Two participants were lost following baseline data collection. Thus, 149 participants completed the follow-up questionnaire. Among them, 144 were followed up in person and five were followed up by phone. The median age of the participants was 57 years old (IQR=49-67) and the majority of participants were female (70%), married (62%), literate (97%), had completed primary or secondary school (81%) and identified as Sri Lankan Tamil (97%) ( Table 1). On average, participants were diagnosed with AF 13 years prior to data collection (mean=156.7 months, SD=104.2; median=159 months, IQR=80-280) ( Table 2).

**Table 1.** Baseline characteristics of participants.

Demographics	Total sample (n=151)
Age, years	
Median (IQR)	57 (49-67)
< 40	10 (6.6)
40 to 50	35 (23.2)
51 to 60	40 (26.5)
61 to 70	47 (31.1)
71 to 80	17 (11.3)
81 +	2 (1.3)
Sex	
Female	105 (69.5)
Male	46 (30.5)
Marital status	
Married	94 (62.3)
Widowed	31 (20.5)
Single	19 (12.6)
Divorced	7 (4.6)
Ethnicity	
Sri Lankan Tamil	146 (96.6)
Moor	1 (0.7)
Indian Tamil	1 (0.7)
Burgher	3 (2.0)
Education	
Did not complete primary school	22 (14.6)
Completed primary school	39 (25.8)
Completed secondary education	84 (55.6)
Holds undergraduate degree	6 (4.0)
Holds postgraduate degree	0 (0)
Literacy	
Illiterate (unable to read or write)	4 (2.6)
literate	147 (97.4)
Employment status	
Housewife	87 (57.6)
Employed	28 (18.5)
Unable to work	20 (13.2)
Retired	12 (7.9)
Cannot find suitable job	1 (0.7)
Does not want to work	1 (0.7)
Student	0 (0)
Other	2 (1.3)

**Table 2.** Baseline questionnaire results.

Question text	Response data N (%)
When were you first diagnosed with this health problem (irregular heartbeats)?	
Months [mean (SD)]	156.7 (±104.2)
I do not know	2/151 (1)
Missing / unknown	0/151 (0)
Who first diagnosed your heart rhythm problem?	
No one, this is the first time I have presented to anyone with this health problem	0 (0)
Primary care units	0 (0)
This hospital at an outpatient department (tertiary care)	3/151 (2)
This hospital at an inpatient department (tertiary care)	39/151 (26)
This hospital at the A&E department (tertiary care)	57/151 (38)
Other government hospital outpatient department (secondary care)	26/151 (17)
Other government hospital inpatient department (secondary care)	0/151 (0)
Private family doctor (small clinic)	17/151 (11)
Private hospital outpatient department	7/151 (5)
Private hospital inpatient department	0/151 (0)
Pharmacist	0/151 (0)
Allopathic medicine	0/151 (0)
Religious or traditional healer	0/151 (0)
Other (diagnosed out of country)	2/151 (1)
Missing / unknown	0/151 (0)
What were you asked to do after leaving from this visit? ^a^	
Come back for a check-up at this outpatient/specialised AF clinic	114/151 (75)
Come back for a check-up at this outpatient/medical clinic	41/151 (27)
Go to private doctor	0/151 (0)
Go to another hospital doctor	1/151 (1)
Urgent admission to hospital due to bleeding	5/151 (3)
Urgent admission to hospital due to stroke	0/151 (0)
Get some new medication	0/151 (0)
Continue with current medication	110/151 (73)
Blood thinning/INR test/clinic	119/151 (79)
Make a lifestyle or behavioural change	5/151 (3)
I do not know	0/151 (0)
Other (take a thyroid stimulating hormone test)	1/151 (1)
Missing / unknown	0/151 (0)
Are you on blood thinning medication now or were you advised to start a blood thinning medication or visit a clinic to get a test for blood thinning medicine?	
Yes	150/151 (99)
No and not advised to start blood thinning medicine or go for a test (contraindicated with other medications or conditions)	1/151 (1)
I do not know	0/151 (0)
Missing /unknown	0/151 (0)
Are you taking any other medications for the treatment of your heart rhythm problem/AF?	
Yes [go to next question]	121/151 (80)
No, he said I do not need anything [skip next question]	30/151 (20)
I do not know [skip next question]	0/151 (0)
Missing /unknown [skip next question]	0/151 (0)
If you are taking other medications for the treatment of your heart rhythm problem/AF, what kind of medication is it? ^a^	
Aspirin	8/121 (5)
Amiodarone (Ancoron)	7/121 (5)
Digoxin	75/121 (50)
Beta-Blocker (e.g. Atenolol, propranolol, carvedilol)	46/121 (30)
Other (Calcium channel blocker)	16/121 (10)
I do not know	0/121 (0)
Missing / unknown	0/121 (0)

^a^ Participants were able to choose more than one option for this question; the proportions may not add up to 100%.

### Patient pathway

**Figure 2.**
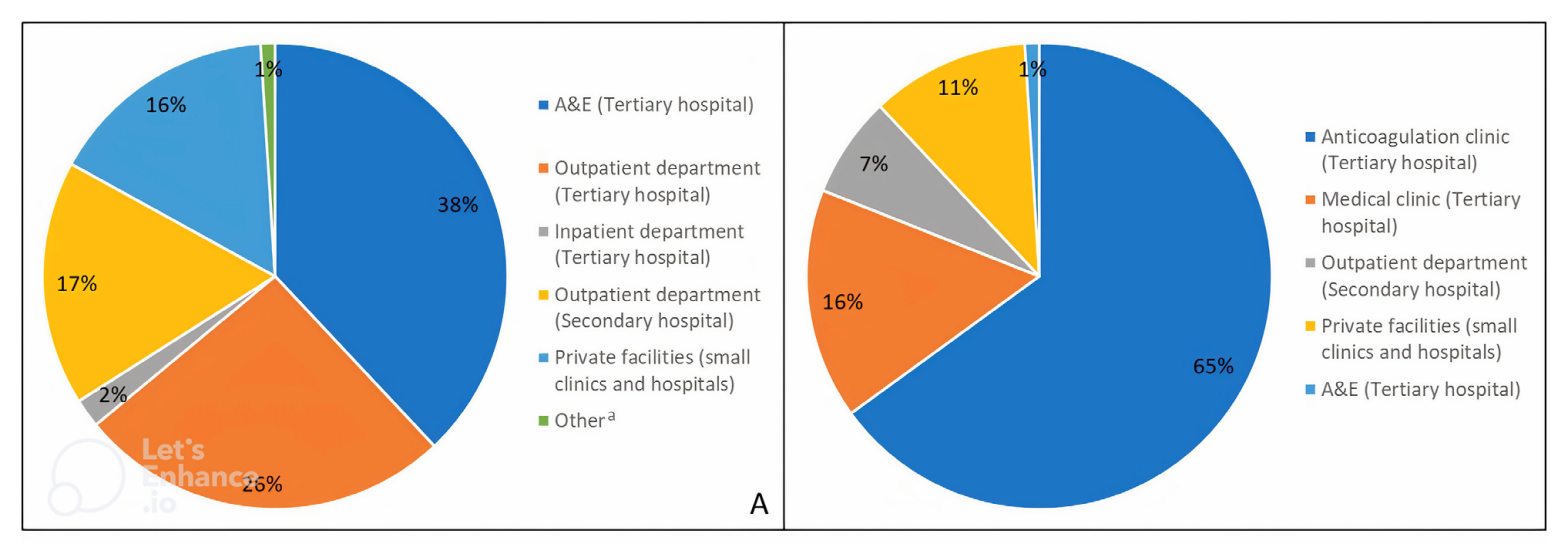
( **A**) Place of AF diagnosis and ( **B**) place of AF care (denominator = 184 *i.e.*, number of total AF care visits since baseline). ^a^ Other includes participants that were diagnosed in a different country. AF, atrial fibrillation.

Most participants (99/151, 66%) were diagnosed in the Jaffna Teaching Hospital; among them more than half (57/99, 58%) were diagnosed in the A&E, accounting for 38% (57/151) of the total sample ( Figure 2). Of those diagnosed at Jaffna Teaching Hospital, 39% (39/99) were diagnosed in the inpatient department, whereas only 3% (3/99) were diagnosed in the outpatient department, accounting for 26% (39/151) and 2% (3/151) of the total sample, respectively. No one was diagnosed with AF in an inpatient department within a base or district general hospital ( *i.e.*, secondary care); however, 17% (26/151) were diagnosed in an outpatient department located in a secondary healthcare facility. One in six (24/151, 16%) participants were diagnosed with AF in a private healthcare facility and 1% (2/151) was diagnosed outside the country.

Most participants (114/151, 75%) were advised to attend the anticoagulation clinic for follow-up care ( Table 3). Indeed, only 3% (4/149) of participants had no follow-up care during the study period. The remaining participants had a total of 184 AF-related care visits with healthcare professionals, averaging 1.3 visits per participant. Two healthcare visits (1%) were to A&E due to a transient ischaemic attack. Most other healthcare visits for AF care were at the anticoagulation clinic (119/184, 65%), followed by 16% (30/184) at the medical clinic, 7% (12/184) at a secondary care outpatient department and 11% (21/184) at a private healthcare facility ( Figure 2). At the time of study follow-up, the average number of days since the participants’ last visit to the anticoagulation and medical clinic was 57.5 days (SD=23.9) and 39.6 days (SD=16.7), respectively. This was higher for those that had visited secondary (82.5; SD=17.5) or private healthcare facilities (81.0; SD=18.6 for private outpatient department and 75.0; SD=23.2 for private small clinic) ( Table 3).

**Table 3.** Follow up questionnaire results including COVID-19 questions.

Question text	Response data N (%)
Have you consulted anyone about your AF/irregular heart rhythm since you were last interviewed?	
Yes	145/149 (97)
No	4/149 (3)
I do not know	0/149 (0)
Missing / unknown	0/149 (0)
Who have you seen about your AF/irregular heart rhythm since the last interview? ^a, b^	
Specialised AF clinic (tertiary care)	119/ 184 (65)
Specialised medical clinic (tertiary care)	30/184 (16)
Was admitted to hospital due to stroke/TIA/Thromboembolism (tertiary care)	2/184 (1)
Was admitted to hospital due to bleeding (tertiary care)	0/184 (0)
Other government hospital outpatient department (secondary care)	12/184 (7)
Other government hospital inpatient department (secondary care)	0/184 (0)
Private doctor (small clinic)	21/184 (11)
Private hospital outpatient department	0/184 (0)
Private hospital inpatient department	0/184 (0)
Pharmacist	0/184 (0)
Allopathic medicine	0/184 (0)
Religious or traditional health	0/184 (0)
Other	0/184 (0)
Missing / unknown	0/184 (0)
How many days since your last visit to the specialised AF clinic for your AF/irregular heart rhythm? [mean (SD)]	
Specialised AF clinic (tertiary care)	39.6 (± 16.7)
Specialised medical clinic (tertiary care)	57.5 (± 23.9)
Was admitted to hospital due to stroke/TIA/Thromboembolism (tertiary care)	0 (0)
Was admitted to hospital due to bleeding (tertiary care)	0 (0)
Other government hospital outpatient department (secondary care)	82.5 (± 17.5)
Other government hospital inpatient department (secondary care)	0 (0)
Private doctor (small clinic)	75.0 (± 23.2)
Private hospital outpatient department	81.0 (± 18.6)
Private hospital inpatient department	0 (0)
Pharmacist	0 (0)
Allopathic medicine	0 (0)
Religious or traditional health	0 (0)
Other	0 (0)
Missing / unknown	0 (0)
If you are on blood thinning medication, have you had blood tests to check your blood thinning medicine since you were last interviewed?	
No I was not referred for a blood test when I left specialised AF clinic	4/149 (3)
No, I was referred for a blood test when I left specialised AF clinic but did not go	7/149 (5)
Yes	138/149 (92)
I do not know	0/149 (0)
Missing / unknown	0/149 (0)
Has the blood thinning medication been easy to get since you were last interveiwed?	
Yes, I was given it at the specialised AF clinic	114/149 (77)
Yes, it was easy to get in a pharmacy	8/149 (5)
No I could not find it or was very hard to find	25/149 (17)
I do not know	0/149 (0)
Missing / unknown	0/149 (0)
Has the blood thinning medication been affordable since you were last interviewed?	
Yes, I was given it at the specialised AF clinic	109/149 (74)
Yes, I was given it at the specialised medical clinic	26/149 (17)
Yes, it was affordable to get in pharmacy/chemist	0/149 (0)
No, it was expensive for me	14/149 (9)
I do not know	0/149 (0)
Missing / unknown	0/149 (0)
During the time of lockdown/strict restrictions, were your visits to the hospital/clinic for your AF/irregular heart rhythm impacted by Covid- 19?	
Yes	58/149 (39)
No	91/149 (61)
I did not have visits to the hospital/clinic before the pandemic so cannot say	0/149 (0)
I did not need visits to the hospital/clinic during this time period so cannot say	0/149 (0)
During the time of lockdown/strict restrictions how were your visits to the hospital/clinic for your AF/irregular heart rhythm impacted from Covid-19?	
I had less visits than usual	33/58 (57)
I had more visits than usual	0/58 (0)
I had to visit a different hospital/clinic than usual	5/58 (9)
I had virtual visits with the doctor/nurse (for example on the phone)	4/58 (7)
My visits were quicker than usual	0/58 (0)
My visits were longer than usual, but I was able to see a doctor/ nurse most of the time	0/58 (0)
My visits were longer than usual, and I was not able to see a doctor/ nurse most of the time	9/58 (16)
I do not know	7/58 (12)
Other	0/58 (0)
During the time of lockdown/strict restrictions, why were your visits to the hospital/clinic for your AF/irregular heart rhythm impacted by Covid-19?	
I was scared of getting Covid-19 or I was shielding, isolating or quarantining	22/58 (38)
My usual clinic closed or reduced their opening hours	6/58 (10)
Local restrictions were enforced such as curfews or lockdowns so I could not visit the hospital/clinic	8/58 (14)
The doctor/nurse I usually see was no longer available	5/58 (9)
Transportation became a problem because public transit was less available/not available at all	6/58 (10)
Transportation became a problem because the person that usually took me was scared of getting Covid-19 or they were shielding/ isolating/quarantining	1/58 (2)
I do not know	10/58 (17)
Other	0/58 (0)
During the time of lockdown/strict restrictions, were your medications for your AF/irregular heart rhythm impacted by Covid-19?	
Yes	58/149 (39)
No	91/149 (61)
I did not have visits to the hospital/clinic before the pandemic so cannot say	0/149 (0)
I did not need visits to the hospital/clinic during this time period so cannot say	0/149 (0)
During the time of lockdown/strict restrictions, how were your medications for your AF/irregular heart rhythm impacted by Covid-19?	
I was not been able to get prescriptions for my medicine from my doctor/nurse	10/58 (17)
My prescriptions were delayed from my doctor/nurse	21/58 (36)
My medication was not available at the pharmacy	13/58 (22)
I had to go to a different place to pick up my medication	0/58 (0)
My medications had to be delivered to me by ambulance, post or public health staff	14/58 (24)
I do not know	0/58 (0)
Other	0/58 (0)
During the time of lockdown/strict restrictions, why were your medications for your AF/irregular heart rhythm impacted by Covid-19?	
I was scared of getting Covid-19 or I was shielding, isolating or quarantining	23/58 (40)
Where I usually picked up my medication was closed or reduced their hours	12/58 (21)
The doctor/nurse was difficult or unavailable to contact for my prescription	7/58 (12)
Transportation became a problem because public transit was less available/not available at all	6/58 (10)
Transportation became a problem because the person that usually took me was scared of getting Covid-19 or they were shielding/ isolating/quarantining	1/58 (2)
I do not know	9/58 (16)
Other	0/58 (0)
During the time of lockdown/strict restrictions, were your tests for your AF/irregular heart rhythm impacted by Covid-19?	
Yes	58/149 (39)
No	91/149 (61)
I did not have visits to the hospital/clinic before the pandemic so cannot say	0/149 (0)
I did not need visits to the hospital/clinic during this time period so cannot say	0/149 (0)
During the time of lockdown/strict restrictions, how were your tests for your AF/irregular heart rhythm impacted by Covid-19?	
I have taken tests with longer intervals than I used to	51/58 (88)
I have stopped taking my tests	6/58 (10)
I do not know	1/58 (2)
Other	0/58 (0)
During the time of lockdown/strict restrictions, why were your tests for your AF/heart rhythm impacted by Covid-19?	
I was scared of getting Covid-19 or I was shielding, isolating or quarantining	23/58 (40)
Where I usually went for tests was closed or had reduced their opening hours	12/58 (21)
Local restrictions were enforced such as curfews or lockdowns so I could not go for tests	7/58 (12)
Transportation became a problem because public transit was less available/not available at all	6/58 (10)
Transportation became a problem because the person that usually took me was scared of getting Covid-19 or they were shielding/ isolating/quarantining	1/58 (2)
I stopped taking my medication and no longer needed tests	6/58 (10)
I do not know	9/58 (16)
Other	0/58 (0)

^a^ Participants were able to choose more than one option for this question; the proportions may not add up to 100%. ^b^ The denominator is from the total number of responses instead of total number of participants.

### Medication and INR tests

Nearly all (150/151, 99%) participants were on blood thinning medication at baseline ( Table 3). The one participant not on treatment was contraindicated due to their comorbid conditions. A total of 80% (121/151) of participants were taking rate or rhythm control medications. This included digoxin (75/121, 50%), beta blocker (46/121, 30%), calcium channel blocker (16/121, 10%), aspirin (8/121, 5%) and amiodarone (7/121, 5%) medication. At follow-up, 92% (138/149) of participants said they had taken an INR test since baseline. Although the majority (122/149, 82%) of participants stated that they can easily get warfarin from Jaffna Teaching Hospital or pharmacy, 17% (25/149) said it is not easy to access warfarin, likely due to limitations caused by the pandemic.

### Impact from the COVID-19 pandemic

Overall, 39% of participants (58/149) said that their visits to the specialised clinics, getting medication and INR tests were adversely impacted during the COVID-19 pandemic ( Table 3). Most of these participants had less visits than usual (33/58, 57%) and some had longer (total time) healthcare visits (9/58, 16%). Similarly, the interval between INR tests became longer (51/58, 88%) and some stopped taking tests during this time because they stopped taking their medication (6/58, 10%). Prescriptions for anticoagulation medication were not attainable for 17% (10/58) of participants and they were delayed for 36% (21/58) of participants. A further 22% (13/58) of participants said the medication was not available from the private pharmacies during this time and 24% (14/58) had their medication delivered to them by ambulance, public health staff or post.

Many participants expressed that the pandemic adversely impacted their visits (22/58, 38%), medication (23/58, 40%) and INR tests (23/58, 40%) because they feared getting COVID-19 or they were shielding, isolating or quarantining. Limited available public transportation, reduced hours or closure of healthcare facilities, enforcement of curfews or lockdowns and doctors being difficult or unavailable to contact (especially in the medical clinics) were additional reasons participants said the pandemic adversely impacted their clinic visits and access to anticoagulation medication and INR tests.

## Discussion

This study investigated key aspects of the existing AF care pathway in Sri Lanka to identify gaps in care that would require improvements to reduce AF-related morbidity and mortality and strengths of current care that could be utilized for improving the AF care pathway. We found that primary care plays no part in diagnosis or management of AF, creating inefficiencies in early diagnosis that lead to patients presenting with a medical crisis at emergency departments and admitted to inpatient wards. Such gaps in care have major clinical and health system implications and should be addressed through policy and health systems improvements. Most patients were taking warfarin and rate and rhythm control medications and had taken at least one INR test within the study period. The outpatient departments were highly utilized for follow-up care; however, patients had less healthcare visits than what is advised by guidelines ^28^, which can partly be explained by the adverse impacts of the COVID-19 pandemic. Alternative medication delivery options during the pandemic were experienced by 24% of participants, allowing for learnings on how to mitigate disruptions to continuity of care in future crises by health system strengthening.

This study revealed that in the Northern Province of Sri Lanka, AF diagnosis occurs mostly in A&E or on the inpatient wards of Jaffna Teaching Hospital ^23^. This leads to the supposition that many patients are diagnosed following a major AF-related symptom or crisis ( *e.g.*, stroke). If we assume that an estimated 10 to 40% of patients with AF are asymptomatic based on evidence from other settings ^29, 30^, there may be a considerable number of undiagnosed AF in the community; a concerning, but not uncommon reality in an LMIC setting ^31^. To enable early diagnosis of symptomatic and silent AF before a medical crisis ^32^ and reduce poor prognosis and high healthcare costs ^12^, the primary care system must be strengthened. This may require a combination of training for healthcare professionals, reliable access to diagnostic equipment, and increased manpower at primary care level. Additionally, patient education ^32^ on AF (how common it is, how it can be effectively managed and how to conduct self-pulse checking), related symptoms (shortness in breath, excessive tiredness, palpitation) and risk factors (age, obesity, hypertension, diabetes and cardiovascular risks) is important to ensure patients can be aware of how to prevent their risk and seek primary healthcare if any related symptoms occur or if they notice heartbeat irregularities through self-pulse checking ^33^.

The use of rate and rhythm control medication and monthly monitoring of INR is a crucial part of AF care to ensure efficacy and safety ^34, 35^. The high use of rate and rhythm control medications in our study (80%) is in line with existing evidence from 35 European countries ^35^ and nearly all participants in our study received INR tests within the study period. However, guidelines recommend monthly INR tests for patients on warfarin ^26^, but during our 3-month study period, participants had on average 1.3 healthcare visits for AF follow-up care. The reasons for this could be multi-factorial, including reasons due to the COVID-19 pandemic. Another plausible reason may be due to the limited access to INR tests. Our previous research found that patients with AF must travel far distances to receive care at the Jaffna Teaching Hospital and is a barrier to optimal care (in pre-pandemic times), particularly for rural inhabitants ^36^. Similar findings have been highlighted in our research in Brazil, indicating that access to INR tests is an issue in other LMICs ^37^.

Local lockdown and restrictions were imposed to control the spread of the COVID-19 pandemic, which impacted the care pathway of patients with AF, leading to another potential reason for the fewer visits to the anticoagulation clinic. Indeed, many participants stated that they had fewer healthcare visits and longer INR intervals during the pandemic, which were partially due to a lack of public transportation. Though, the health system was creative and succeeded in a quarter of cases to ensure partial continuity of care during the pandemic by providing delivery options for medications through post, ambulance services and public health professionals ^38^. These innovations of delivering medications through various modes may prove beneficial to those that live far from the hospital or those with physical disabilities, thereby overcoming the barrier of having to travel long distances for care and facilitate the implementation of the ABC pathway ^6^. Further research should investigate the possible positive impacts ( *e.g.*, costs, clinical outcomes and patient satisfaction) of offering these delivery options for medications and providing INR tests closer to home in Sri Lanka and other LMICs that likely suffer from the same barriers.

### Strengths and limitations

This study was conducted at the only tertiary hospital in the Northern Province that has cardiologists and can conduct 12-lead echocardiogram tests. Thus, most patients with AF in the province that retain AF care from government facilities, must visit Jaffna Teaching Hospital for care ^36^. Our sample is therefore largely representative of all patients with AF receiving government-funded care in the region. However, the study was subject to a few limitations. The required sample size for the study was not met. This was mainly due to recruitment limitations as described in a previous publication ^23^; although, lost to follow-up was minimal and this is a purely descriptive study. Therefore, these limitations do not largely impact the conclusions we could draw from our sample regarding our objectives particularly since one of our key findings is that primary care was absent in the diagnosis and management of AF. Most participants were diagnosed with AF more than 10 years prior to baseline and therefore the place of diagnosis may not reflect current practice and therefore should be interpreted with caution. As recruitment took place in a public hospital setting, this study does not include patients that were diagnosed but not retained in care or those that receive care entirely in private facilities. During the COVID-19 pandemic, the country experienced a nationwide lockdown and restrictions were imposed on in-country travel and curfews were enforced ^38^. As reported by participants, this impacted the AF pathway in terms of receiving medication, visiting the doctor and receiving INR tests; thus, potentially impacting our understanding and interpretation of the ‘usual’ AF care pathway ^6^ ( *i.e.*, the pathway prior to the pandemic). However, the anticoagulation clinic functioned as usual during the lockdown period where many patients visited for care. Additionally, given this is the first study to investigate the AF care pathway in a South Asian country and one of the few to be conducted in any LMIC, the results of our study are nonetheless a critical start to understanding how and where patients with AF go for care, what areas of the pathway need improvements and what needs to be prioritized in future AF research in LMICs.

In conclusion, our findings highlight that AF diagnoses mostly occur in the A&E or inpatient department in the Northern Provence of Sri Lanka. Whilst most patients retained in care at the Jaffna Teaching Hospital (the only facility with specialist AF care) ^36^ are on anticoagulants and rate and rhythm control medication and receive routine INR tests, early diagnosis in primary care facilities must be prioritized in Sri Lanka to provide life-saving preventative medications for reducing AF-related strokes and achieve the first step within the ABC pathway. The frequency of follow-up care visits was fewer than guideline recommendations, which in part was due to the COVID-19 pandemic but could also be due to the limited access to INR tests, a barrier ^38^ likely shared by other LMICs. Learnings from the COVID-19 pandemic can be used to provide a range of delivery options closer to patients’ home for medications and INR tests to improve access to care and reduce disruptions to continuity of care during and beyond a time of crisis. Strengthening primary and community-based care would be conducive for improving the early diagnosis of and continuity of care for AF in Sri Lanka and other LMICsr ^39^; thus, could contribute to the global Sustainable development Goal 3 of reducing premature death caused by NCDs.

## Data Availability

As the AF pathway dataset contains sensitive health data set as well as the other sensitive personal information, the raw data set cannot be published at the individual level. The data set is available at Open Science Framework open repository ( https://doi.org/10.17605/OSF.IO/UHT7D) and available under restricted access and can be obtained by submitting a request directly to the corresponding author ( kumarendran@univ.jfn.ac.lk).
