# Supplementary material for: The pathway to diagnosis and follow-up care for atrial fibrillation in Sri Lanka: a descriptive longitudinal study
Source: NIHR Open Res. 2026 Feb 23;3:63. Originally published 2023 Nov 24. [Version 3] doi: 10.3310/nihropenres.13497.3 (PMC11320028; doi:10.3310/nihropenres.13497.3)
Supplement: Peer review history

# Reviewer report: Reviewer response for version 2

**DOI:** 10.3310/nihropenres.14800.r32195

**Published:** 2024-07-09

**Authors:** Raif Kılıç

**Affiliations:** 1 Memorial Diyarbakır Hospital, Cermik, Diyarbakır, Turkey

In this manuscript, the authors examined “identifying the AF care pathway in the Northern Province of Sri Lanka and how the COVID-19 pandemic has affected the care pathway.” In this respect, I congratulate the authors and find this manuscript important. However, here I have some revision suggestions for the authors;

 -The introduction is quite long and detailed. Main focus points can be made clear and expressed more succinctly. The introduction can be kept shorter to attract the reader's attention and help them focus more quickly on the main topic of the article.

 -Some paragraphs in the introduction appear to contain repetitive or similar information to each other. These paragraphs can be combined in a more compact and concise manner.

 - It was stated that patient partners were not involved in the design and conduct of the study. Lack of patient participation may limit the impact and validity of the study's results to real-world applications. It is recommended that this be stated in the limitations section.

 - How the diagnosis of AF is confirmed and the definition and criteria of “arrhythmia likely to be AF” can be explained in more detail.

 - It was stated that the study was conducted only in the Jaffna region. This may limit the generalizability of the results to other regions across Sri Lanka. It is recommended that this topic be added to the limitations section.

 - The policy and health system improvements suggested in the discussion section may be more specific. What policy changes are being proposed and how can the feasibility and effectiveness of these recommendations be evaluated?

 - To increase the evidence and quality of the study, you can cite the reference below;

Is the work clearly and accurately presented and does it cite the current literature?

Partly

If applicable, is the statistical analysis and its interpretation appropriate?

Partly

Are all the source data underlying the results available to ensure full reproducibility?

Partly

Is the study design appropriate and is the work technically sound?

Yes

Are the conclusions drawn adequately supported by the results?

Yes

Are sufficient details of methods and analysis provided to allow replication by others?

Yes

Reviewer Expertise:

arrhythmiaheart failureinterventional cardiology

I confirm that I have read this submission and believe that I have an appropriate level of expertise to confirm that it is of an acceptable scientific standard, however I have significant reservations, as outlined above.

## References

[1] : The effect of treatment strategy on long-term follow-up results in patients with nonvalvular atrial fibrillation in Turkey: AFTER-2 subgroup analysis. *Aging Clin Exp Res* .2023;35(8) : 10.1007/s40520-023-02467-y 1695-1704 10.1007/s40520-023-02467-y 37329474

# Response

**Published:** 2025-12-02

**Authors:** Sheron Antony

**Affiliations:** Community and Family Medicine, University of Jaffna, Jaffna, Northern Province, Sri Lanka

Reviewer (3): Raif Kılıç, Memorial Diyarbakır Hospital, Cermik, Diyarbakır, Turkey

 In this manuscript, the authors examined “identifying the AF care pathway in the Northern Province of Sri Lanka and how the COVID-19 pandemic has affected the care pathway.” In this respect, I congratulate the authors and find this manuscript important. However, here I have some revision suggestions for the authors;

 Dear Raif Kılıç,

 Thank you for you review for our manuscript titled “The pathway to diagnosis and follow-up care for atrial fibrillation in Sri Lanka: a descriptive longitudinal study”. We have addressed each comment in a point-by-point format below and have modified the manuscript accordingly. We have uploaded the updated manuscript with tracked changes.

**Comment 1:**

 The introduction is quite long and detailed. Main focus points can be made clear and expressed more succinctly. The introduction can be kept shorter to attract the reader's attention and help them focus more quickly on the main topic of the article. Some paragraphs in the introduction appear to contain repetitive or similar information to each other. These paragraphs can be combined in a more compact and concise manner.

**Response:**

 We thank the reviewer for this insightful comment. We have carefully revised the introduction to enhance clarity and conciseness by removing repetitive and non-essential content. Nevertheless, we have intentionally retained key contextual details related to the evidence-based atrial fibrillation (AF) management framework, particularly the ABC pathway, and its implementation disparity between high-income and low- and middle-income countries such as Sri Lanka. We consider this information crucial to justify the study’s rationale and to position our work within the broader global effort to improve AF care pathway.

**Comment 2:**

 It was stated that patient partners were not involved in the design and conduct of the study. Lack of patient participation may limit the impact and validity of the study's results to real-world applications. It is recommended that this be stated in the limitations section.

**Response:**

 We agree this is a limitation and we have added this in the manuscript (see below).

 “Further, patient and public involvement was not incorporated in the design or conduct of this study. The absence of direct patient engagement may limit the contextual relevance and transferability of the findings to routine clinical practice.

**Comment 3:**

 How the diagnosis of AF is confirmed and the definition and criteria of “arrhythmia likely to be AF” can be explained in more detail.

**Response:**

 We based our eligibility criteria on self-report by the patient and notes from their clinic book if and when this was available. We have clarified this within the amended manuscript (see below).

 “Adults (≥18 years) with a confirmed diagnosis of AF (who were already diagnosed) or an arrhythmia likely to be AF (presented with an irregular heart rhythm on ECG, clinical assessment, or based on symptoms, suggestive of AF) who were visiting any of the included healthcare facilities for AF care and spoke the local language (Tamil) were considered eligible.”

**Comment 4:**

 It was stated that the study was conducted only in the Jaffna region. This may limit the generalizability of the results to other regions across Sri Lanka. It is recommended that this topic be added to the limitations section.

**Response:**

 We agree that the regional focus of the study may limit the generalisability of the findings. We have added a statement in the limitations section acknowledging that the findings from this study may not be generalisable to other regions of Sri Lanka.

 “Also, the study was limited to participants from the Northern region, which may restrict the generalisability of the results to other areas of Sri Lanka that differ in population characteristics, healthcare access, or AF screening practices.”

**Comment 5:**

 The policy and health system improvements suggested in the discussion section may be more specific. What policy changes are being proposed and how can the feasibility and effectiveness of these recommendations be evaluated?

**Response:**

 We appreciate this feedback. We have mentioned various recommendations for future research and potential policy improvements (see below). We are happy to expand on any of these that you feel require further explanation or details.

 “To enable early diagnosis of symptomatic and silent AF before a medical crisis and reduce poor prognosis and high healthcare costs, the primary care system must be strengthened. This may require a combination of training for healthcare professionals, reliable access to diagnostic equipment, and increased manpower at primary care level, particularly on how to screen and manage the different types of AF and comorbidities such as coronary artery disease due to the complex relationship between the two conditions. Additionally, patient education on AF (how common it is, how it can be effectively managed and how to conduct self-pulse checking), related symptoms (shortness in breath, excessive tiredness, palpitation) and risk factors (age, obesity, hypertension, diabetes and cardiovascular risks) is important to ensure patients can be aware of how to prevent their risk and seek primary healthcare if any related symptoms occur or if they notice heartbeat irregularities through self-pulse checking.”

 “NOACs can be an effective alternative to warfarin especially in LMICs where INR facilities are limited, however, the costs of NOACs are hindering their introduction in Sri Lanka and many other LMICs and this must be addressed.”

 “Further research should investigate the impacts of offering these delivery options for medications and providing INR tests closer to home on health system and patient cost, clinical outcomes and patient satisfaction in Sri Lanka and other LMICs that likely suffer from the same barriers.”

**Comment 6:**

 To increase the evidence and quality of the study, you can cite the reference below;

**Response: **We agree that it is relevant to our study and it is now cited in the revised manuscript to strengthen the evidence base and enhance the quality of the discussion.

 Kılıç R, Güzel T, Aktan A, Demir M, et al.: The effect of treatment strategy on long-term follow-up results in patients with nonvalvular atrial fibrillation in Turkey: AFTER-2 subgroup analysis. *Aging Clin Exp Res*. 2023; **35** (8): 1695-1704

# Reviewer report: Reviewer response for version 2

**DOI:** 10.3310/nihropenres.14800.r32003

**Published:** 2024-06-20

**Authors:** Luigina Guasti

**Affiliations:** 1 University of Insubria, Varese, Italy

The revised paper is suitable for indexing.

Is the work clearly and accurately presented and does it cite the current literature?

Partly

If applicable, is the statistical analysis and its interpretation appropriate?

Yes

Are all the source data underlying the results available to ensure full reproducibility?

Partly

Is the study design appropriate and is the work technically sound?

Partly

Are the conclusions drawn adequately supported by the results?

Yes

Are sufficient details of methods and analysis provided to allow replication by others?

Yes

Reviewer Expertise:

geriatric cardiology, dyslipidemias

I confirm that I have read this submission and believe that I have an appropriate level of expertise to confirm that it is of an acceptable scientific standard.

# Reviewer report: Reviewer response for version 1

**DOI:** 10.3310/nihropenres.14647.r30890

**Published:** 2024-01-04

**Authors:** Luigina Guasti

**Affiliations:** 1 University of Insubria, Varese, Italy

- In Abstract the authors state that "We aimed to identify the AF patient pathway in Northern Province, Sri Lanka and to determine how the COVID-19 pandemic impacted AF care". If so, this should be focussed on in the title.

 - In abstract ( ".... regular monitoring when treated with warfarin..." ) conclusion ("....frequency of blood tests was lower...") and throughout the text the authors refer to VKA. However, NOACs are widely used as OAC, at least in non LMICs. This aspect should be highlighted in the various sections of the text. 

 - in study design the authors cite reference #23 as the main paper (multicountry study) where the results on the 152 patients from Sri Lanka were reported. That paper indicated potential bias in recruting (70% of females from the sample in Sri Lanka who may show a different attitude towards the proposed care). This aspect should be discussed.

Is the work clearly and accurately presented and does it cite the current literature?

Partly

If applicable, is the statistical analysis and its interpretation appropriate?

Yes

Are all the source data underlying the results available to ensure full reproducibility?

Partly

Is the study design appropriate and is the work technically sound?

Partly

Are the conclusions drawn adequately supported by the results?

Yes

Are sufficient details of methods and analysis provided to allow replication by others?

Yes

Reviewer Expertise:

geriatric cardiology, dyslipidemias

I confirm that I have read this submission and believe that I have an appropriate level of expertise to confirm that it is of an acceptable scientific standard, however I have significant reservations, as outlined above.

# Response

**Published:** 2024-05-29

**Authors:** Sheron Antony

**Affiliations:** Community and Family Medicine, University of Jaffna, Jaffna, Northern Province, Sri Lanka

**Reviewer 2 (Luigina Guasti) comments:**

 Thank you for you review for our manuscript titled “The pathway to diagnosis and follow-up care for atrial fibrillation in Sri Lanka: a descriptive longitudinal study”. We have addressed each comment in a point-by-point format below and have modified the manuscript accordingly. We have uploaded the updated manuscript with tracked changes.

 Yours sincerely,

 Mr V A Sheron

**Comment 1: **In Abstract the authors state that "We aimed to identify the AF patient pathway in Northern Province, Sri Lanka and to determine how the COVID-19 pandemic impacted AF care". If so, this should be focussed on in the title.

**Response: **Thank you for the suggestions, we have considered the suggestion and modified the title as “Identifying the care pathway for atrial fibrillation in Sri Lanka and the impact of the COVID-19 pandemic on the care pathway: a descriptive longitudinal study”

**Comment 2: **In abstract ( ".... regular monitoring when treated with warfarin..." ) conclusion ("....frequency of blood tests was lower...") and throughout the text the authors refer to VKA. However, NOACs are widely used as OAC, at least in non LMICs. This aspect should be highlighted in the various sections of the text.

**Response: **We agree this is an important point to make. We have amended the text to ensure this is clear (see below). Please note that whilst NOAC usage is increasing in LMIC settings, warfarin is the only available OAC for AF management offered under the Sri Lankan public health system.

 “On the other hand, Novel oral anticoagulants (NOAC) do not require as much monitoring as warfarin and could be hugely advantageous for LMICs given the barriers of accessing healthcare services and INR tests. Whilst recent studies have found an increase in the availability and use of NOACs in LMICs it is still limited in many LMICs, including Sri Lanka where warfarin is the only available OAC within the public health system” ***(Introduction – Paragraph 2)***

**Comment 3: **In study design the authors cite reference #23 as the main paper (multi-country study) where the results on the 152 patients from Sri Lanka were reported. That paper indicated potential bias in recruiting (70% of females from the sample in Sri Lanka who may show a different attitude towards the proposed care). This aspect should be discussion.

**Response: **We agree this was a limitation, and this was already mentioned in the discussion; however, we have added more text to make this limitation clear.

 “The required sample size for the study was not met, a large proportion were female who may differ in terms of healthcare utilisation than males” ***(Strength and limitation – Paragraph 1)***

# Reviewer report: Reviewer response for version 1

**DOI:** 10.3310/nihropenres.14647.r30887

**Published:** 2023-12-22

**Authors:** Akash Batta

**Affiliations:** 1 Dayanand Medical College and Hospital, Ludhiana, India

The index study by the authors reflects the current status of AF care in a LMIC and highlights a very important aspect: Lack of involvement of primary and secondary care in AF diagnoses and management. Their report although not novel, does make a strong statement in favour of improving the primary care through adequate training of doctors and paramedical staff to pick up the largely under-diagnosed pool of AF patients who only present once there has been a MACCE event which otherwise could have been prevented if picked up earlier. The impact of COVID 19 in their district has also been described in regards to AF care.

 however, the paper needs a major revamp and editing by an English expert to improve its overall quality and value to current practice.

 Concerns:-

 1) The abstract should be clearly written. It has been written rather poorly and casually compared to the main text. The first line should be rewritten "Early diagnosis and continuity of care is vital for atrial fibrillation (AF), a major risk factor for stroke that requires regular monitoring when treated with warfarin". Just mention inserted that AF requires treatment with OAC to prevent stroke. 

 2) Clarify what you mean by "AF patient pathway"

 3) Remove  these unnecessary details" recruited from the A&E department and outpatient clinics located at the Jaffna Teaching Hospital, the only tertiary hospital with cardiologists and 12-lead

 echocardiogram facilities in the Province"

 4) Clearly write the objective and major endpoints of the study.

 5) Add more complications of AF which are clinically relavant like heart failure, myocardial infarction, dementia in addition to stroke alone.

 6) Need to state that warfarin is not synonymous with OAC and recent data even from LMIC shows that NOACS are fast replacing VKAs as the preferred OAC for non valvular AF

 7) The study design should be written as multi-centric rather that multi-country

 8) An important association of AF is coronary artery disease (CAD) which should be described in discussion. This is important as recent evidence indicated a very intricate relationship between AF and CAD which could be utilised at the primary care. Accordingly, one must pay more attention to screening AF in these patents in their primary or secondary care setting. Accordingly, I would strongly suggest addition data on the close link between the two and citing 3-4 recent integrative reviews which highlight this association and guide therapeutic strategies to treat the two diseases.

Is the work clearly and accurately presented and does it cite the current literature?

Partly

If applicable, is the statistical analysis and its interpretation appropriate?

Yes

Are all the source data underlying the results available to ensure full reproducibility?

Yes

Is the study design appropriate and is the work technically sound?

Partly

Are the conclusions drawn adequately supported by the results?

Partly

Are sufficient details of methods and analysis provided to allow replication by others?

Yes

Reviewer Expertise:

My main areas of research are AF pathophysiology and epidemiology. I also have keen interest in clinical cardiology. I seek to explore the association between AF and ischemia at microcirculatory level.

I confirm that I have read this submission and believe that I have an appropriate level of expertise to confirm that it is of an acceptable scientific standard, however I have significant reservations, as outlined above.

## References

[1] : Atrial Fibrillation and Chronic Coronary Ischemia: A Challenging Vicious Circle. *Life (Basel)* .2023;13(6) : 10.3390/life13061370 10.3390/life13061370 37374152 PMC10305229

[2] : Atrial fibrillation and coronary artery disease: An integrative review focusing on therapeutic implications of this relationship. *World J Cardiol* .2023;15(5) : 10.4330/wjc.v15.i5.229 229-243 10.4330/wjc.v15.i5.229 37274376 PMC10237004

[3] : The relationship between atrial fibrillation and coronary artery disease: Understanding common denominators. *Trends Cardiovasc Med* .2022; 10.1016/j.tcm.2022.09.006 10.1016/j.tcm.2022.09.006 36182022

[4] : Patients with atrial fibrillation and coronary artery disease - Double trouble. *Adv Med Sci* .2018;63(1) : 10.1016/j.advms.2017.06.005 30-35 10.1016/j.advms.2017.06.005 28818746

# Response

**Published:** 2024-05-29

**Authors:** Sheron Antony

**Affiliations:** Community and Family Medicine, University of Jaffna, Jaffna, Northern Province, Sri Lanka

Dear Akash Batta,                                                                                            

 Thank you for you review for our manuscript titled “The pathway to diagnosis and follow-up care for atrial fibrillation in Sri Lanka: a descriptive longitudinal study”. We have addressed each comment in a point-by-point format below and have modified the manuscript accordingly. We have uploaded the updated manuscript with tracked changes.

 Yours sincerely,

 Mr V A Sheron

**Reviewer 1 (Akash Batta) comments:**

 The index study by the authors reflects the current status of AF care in a LMIC and highlights a very important aspect: Lack of involvement of primary and secondary care in AF diagnoses and management. Their report although not novel, does make a strong statement in favour of improving the primary care through adequate training of doctors and paramedical staff to pick up the largely under-diagnosed pool of AF patients who only present once there has been a MACCE event which otherwise could have been prevented if picked up earlier. The impact of COVID 19 in their district has also been described in regards to AF care.

 however, the paper needs a major revamp and editing by an English expert to improve its overall quality and value to current practice.

**Response: **Thank you for the positive assessment of the paper. We can confirm that we have read the manuscript thoroughly and have made improvements to the overall quality of the writing. We hope this has elevated the readability of the paper.

**Comment 1: **The abstract should be clearly written. It has been written rather poorly and casually compared to the main text. The first line should be rewritten "Early diagnosis and continuity of care is vital for atrial fibrillation (AF), a major risk factor for stroke that requires regular monitoring when treated with warfarin". Just mention inserted that AF requires treatment with OAC to prevent stroke.

**Response: **Thank you for the suggestions, we have amended the abstract throughout to make it clearer; however, we left the first part of the first sentence given the rationale for our research is related to the need for early diagnosis and continuity of care for AF.

**Comment 2: **Clarify what you mean by "AF patient pathway"

**Response: **We have clarified this in the abstract. For the purpose of our study, we have defined the AF care pathway as comprising the pathway from diagnosis to follow-up/management.

**Comment 3: **Remove these unnecessary details" recruited from the A&E department and outpatient clinics located at the Jaffna Teaching Hospital, the only tertiary hospital with cardiologists and 12-lead echocardiogram facilities in the Province"

**Response: **Thank you for the suggestions, we have removed the unnecessary details as suggested.

**Comment 4: **Clearly write the objective and major endpoints of the study.

**Response: **Thank you, we have amended the abstract to make the aim and outcomes clearer.

**Comment 5: **Add more complications of AF which are clinically relevant like heart failure, myocardial infarction, dementia in addition to stroke alone.

**Response: **We have amended the text in the introduction of the manuscript to include additional clinically relevant complications (see below).

 “AF increases the risk of hospitalisation and stroke, heart failure and myocardial infarction” ***(Introduction – First paragraph)***

**Comment 6: **Need to state that warfarin is not synonymous with OAC and recent data even from LMIC shows that NOACS are fast replacing VKAs as the preferred OAC for non-valvular AF

**Response: **We agree this is an important point to make. We have amended the text to ensure this is clear (see below). Please note that whilst NOAC usage is increasing in LMIC settings, warfarin is the only available OAC for AF management offered under the Sri Lankan public health system.

 “On the other hand, Novel oral anticoagulants (NOAC) do not require as much monitoring as warfarin and could be hugely advantageous for LMICs given the barriers of accessing healthcare services and INR tests. Whilst recent studies have found an increase in the availability and use of NOACs in LMICs it is still limited in many LMICs, including Sri Lanka where warfarin is the only available OAC within the public health system” ***(Introduction – Paragraph 2)***

**Comment 7: **The study design should be written as multi-centric rather that multi-country

**Response: **Thank you for the suggestions, however, we have respectively left the text as ‘multi-country’; we felt multi-centric (whilst technically correct) could also mean several centers within the same country whereas our study was conducted in multiple counties and in multiple centers within two of these countries (Brazil and China).

**Comment 8: **An important association of AF is coronary artery disease (CAD) which should be described in discussion. This is important as recent evidence indicated a very intricate relationship between AF and CAD which could be utilised at the primary care. Accordingly, one must pay more attention to screening AF in these patents in their primary or secondary care setting. Accordingly, I would strongly suggest addition data on the close link between the two and citing 3-4 recent integrative reviews which highlight this association and guide therapeutic strategies to treat the two diseases.

**Response: **Thank you for the suggestions, we have amended the text in the discussion as suggested (see below).

 “This may require a combination of training for healthcare professionals, reliable access to diagnostic equipment, and increased manpower at primary care level, particularly on how to screen and manage the different types of AF and comorbidities such as coronary artery disease due to the complex relationship between the two conditions” ***(Discussion – Paragraph 2)***
